# Difficulties in Accessing Mental Health Services: The Perspective of Users from Cultural Minorities

**DOI:** 10.3390/healthcare14142083

**Published:** 2026-07-12

**Authors:** Antonio Iudici, Giulia Gusella

**Affiliations:** 1 Department of Philosophy, Education, Sociology and Applied Psychology of Padua (FISPPA), University of Padua, 35139 Padua, Italy; 2 Institute of Psychology and Psychotherapy, Mestre, Milano, 35129 Padova, Italy; gusellagiulia1@gmail.com

**Keywords:** ethnology, mental health services, barriers health service, users perspective, inequalities

## Abstract

**Background:** Mental health services face significant challenges in providing equitable care to ethnic minority and migrant populations. Despite the right to healthcare, disparities in service use among minority communities reflect not only practical barriers but also deeper issues of cultural compatibility between patients and health systems. **Aim:** This study aimed to provide a systematic overview of the main difficulties encountered by ethnic minority and migrant people when seeking psychological support from mental health services, with a specific focus on linguistic and communicative barriers and organisational and economic barriers. **Methods:** A scoping review was conducted using the SCOPUS database, searching for peer-reviewed studies focused on the European context. Studies were included if they addressed ethnic minorities’ experiences with mental health services from a user perspective and included primary research of any design. Studies focused exclusively on staff perspectives or not specifically addressing ethnic minorities’ help-seeking were excluded. Twenty studies met the inclusion criteria. A qualitative narrative synthesis was adopted, following PRISMA-ScR guidelines. **Results:** People with an ethnic or migrant background face specific and compounded barriers when seeking mental health support. Two main categories were identified: linguistic and communicative difficulties, including language distance, limited interpreter availability, and the gap between Western biomedical models and cultural frameworks of distress; and organisational and economic obstacles, including poor knowledge of available services, socioeconomic disadvantage, stigma, and institutional distrust. **Discussion:** These barriers are deeply structural and cannot be addressed through awareness campaigns alone. Increased access to services is not inherently beneficial unless accompanied by a fundamental transformation ensuring that care is culturally appropriate, safe, and genuinely responsive to minority communities’ needs. Distrust of mental health services may in part reflect a historically grounded and legitimate response to institutions whose practices have not always served the interests of minority groups. **Conclusions:** Reducing disparities in mental health care requires multi-level intervention, including inclusive policies, training of culturally competent professionals, and a critical rethinking of the models underpinning mental healthcare care. Future research should attend not only to the quantity of service use among minority populations but to the quality, cultural legitimacy, and safety of the care provided.

## 1. Introduction

Groups socially identified as ethnic minorities represent distinct communities perceived as different from the majority population on the basis of cultural, linguistic, religious, or socially constructed characteristics—differences that are the product of historical, social, and political processes rather than biological categories [1,2]. In an increasingly globalised world, migration has contributed to a significant increase in cultural diversity, bringing with it new challenges in healthcare and particularly in mental health services [3]. People from ethnic minorities may face unique experiences of stress, discrimination, marginalisation, and trauma, all of which affect their mental health and treatment needs [4]. It should be noted, however, that the relationship between minority communities and mental health services is not straightforwardly one of unmet need awaiting fulfilment: historically, psychiatric institutions have in some contexts functioned as instruments of social control rather than care, and distrust of such services may in part reflect a historically grounded and rational response to this legacy [5].

Two interconnected sets of barriers have been consistently identified in the literature. The first concerns linguistic and communicative difficulties. Language barriers extend beyond simple lexical misunderstanding: they encompass the gap between Western biomedical models of mental distress and the cultural, spiritual, and collective frameworks through which many minority communities understand and express psychological suffering [6]. Research in the field of bilingualism further suggests that emotions, particularly those associated with early relational experiences and trauma, are primarily encoded and processed in the mother tongue [7,8], raising the possibility that therapy conducted in a second language may hinder the emotional processing central to psychological treatment [9], and that psychiatric care may not be cross-culturally equivalent in ways that go beyond translation [10,11]. The lack of trained professionals in cultural diversity, inconsistent availability of interpreting services, and low mental health literacy within minority communities further compound these difficulties [12,13].

The second set of barriers is organisational and economic in nature. Poverty, precarious employment, and labour market discrimination create a cycle of economic instability that simultaneously increases psychological stress and reduces the resources available to seek treatment [14,15]. Geographical distance from specialised centres, concentration of minority populations in underserved areas, stigma rooted in cultural or religious traditions, and the cumulative burden of trauma related to migration and persecution all further discourage help-seeking and reduce engagement with formal services [13,16,17]. These barriers are deeply structural: many models of psychological care in Western contexts are built around an individualistic view of mental wellbeing that may not be compatible with the collective conceptions prevalent in some cultures [18], underscoring the need for multi-level intervention and genuinely inclusive service design [16,19,20,21].

Understanding how ethnic minorities experience and navigate mental health services requires a theoretical framework sensitive to the social and relational dimensions of human experience. The interactionist perspective, rooted in the works of Blumer [22] and Mead [23] and further developed by contemporary scholars [24,25], posits that social reality is not a fixed entity but a dynamic construct shaped by human interaction and interpretation. Individuals create and negotiate meanings through their social interactions, making our understanding of the world fundamentally a product of shared symbols and collective meaning-making processes [26,27]. This framework is particularly well-suited to examining the complex dynamics that characterise ethnic minorities’ encounters with mental health services, offering insights into how barriers to care are constructed, maintained, and potentially transformed through interaction [28,29,30].

Despite a growing body of literature on mental health inequalities, evidence on the specific barriers experienced by ethnic minorities from a user perspective remains fragmented, and the linguistic and organisational dimensions of these barriers have rarely been examined together in a unified analytical framework. This study therefore addresses the following research questions: What linguistic and communicative barriers do ethnic minorities encounter when accessing mental health services in Europe? What organisational and economic barriers shape their engagement with these services? And to what extent do current services adequately respond to the needs of minority communities?

This study aims to provide a scoping review of the main barriers that ethnic minorities face in accessing mental health services, with a specific focus on linguistic and organisational dimensions in order to highlight the specific needs of minority communities and identify directions for more equitable and culturally responsive mental health care [19,21].

## 2. Methods

### 2.1. Investigation Method: Scoping Review

The scoping review represents a literature research method aimed at mapping key concepts, sources of evidence, and research gaps in a specific field of interest. This approach allows researchers to explore vast and complex areas of study, including diverse types of studies and methodologies. This flexibility is particularly useful when addressing emerging or little-explored themes, where conceptual boundaries may still be blurred [31,32]. It is indeed particularly suitable for exploring emerging or little-known areas. The methodology of the scoping review tends to provide a primarily qualitative synthesis of results and has as a distinctive aspect the iterative nature of the review process, that is, it allows for refining research questions during the review process itself, adapting to emerging information [33,34].

In the specific context of our research objectives, the scoping review has been identified as the most appropriate method for several reasons. Firstly, because the research scope is broad as it includes aspects of discrimination, barriers to service access, and cultural differences in the perception of mental health. This breadth aligns perfectly with the scoping review’s objective of mapping key concepts in this new research area. Furthermore, we are interested in analyzing studies conducted with different methodologies, including qualitative and quantitative studies, which is a specific focus of the scoping review. Certainly, the topic of mental health of ethnic minorities is an evolving research area with limited literature.

In particular, existing evidence indicates that the perception of discrimination, a key element in defining a service as discriminatory, has received limited scholarly attention. Finally, in this field, some gaps in basic research might emerge, such as the possible underrepresentation of ethnic minorities in public mental health services, which is another prerogative of scoping reviews.

### 2.2. Search Strategy, Criteria, and Data Collection

Our scoping review followed a comprehensive and systematic approach to ensure the inclusion of relevant literature [35]. The process was guided by our research questions and established inclusion/exclusion criteria.

The literature search was conducted through the following databases: Scopus, Web of Science, PubMed, and PsycINFO. The search strategy combined controlled vocabulary and free-text terms using Boolean operators (AND, OR) structured as follows: (*ethnic minority* OR *migrant*) AND (*mental health services*) AND (*access barriers* OR *discrimination* OR *inequalities*) AND (*user perspective* OR *users*) AND (*determinants of access*). Key thematic areas addressed by the search included accessibility, barriers to mental health service use, ethnic minority status, and user perspective.

Studies were included if they met the following criteria: focused on the European context, published in English, addressed ethnic minorities’ experiences with mental health services from a user perspective, and included primary research (qualitative, quantitative, or mixed methods). Studies were excluded if they: focused on mental health services staff’s perspective, were written in languages other than English, did not specifically address ethnic minorities seeking help in mental health services. This review was prospectively registered in PROSPERO (CRD420261419368).

The study selection process was conducted in three distinct stages, supervised by the authors of this work: (1) initial screening based on inclusion criteria; (2) evaluation of titles and abstracts; (3) full-text review of potentially relevant articles.

## 3. Results

**Figure 1:**
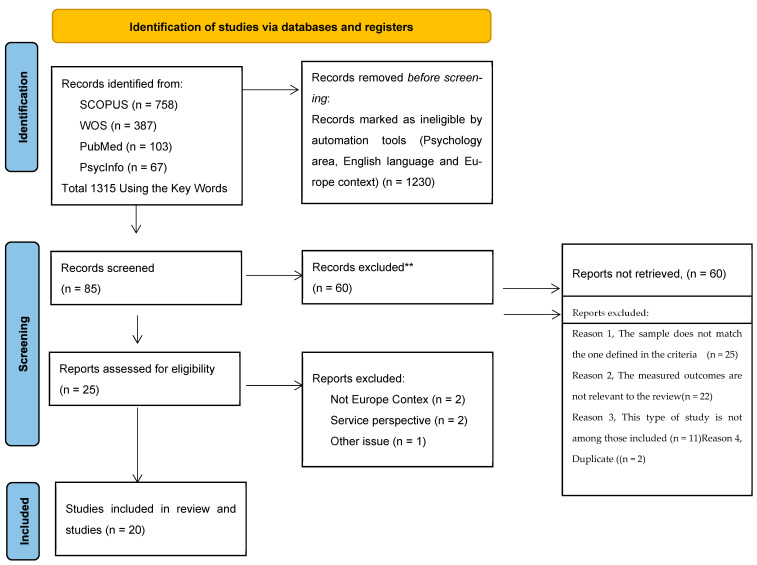
Flow diagram illustrating the processes of literature searches and screening.

From an initial pool of 1315 abstracts, many were immediately excluded as they did not focus on the psychology area. Of the remaining 85 studies considered relevant, 60 were excluded based on our criteria. Ultimately, 20 studies were included in the final analysis (see Figure 1).

The reduction from 1315 to 20 studies reflects the progressive application of inclusion and exclusion criteria across multiple stages. In the first phase of title and abstract screening, duplicates were removed, along with studies that were irrelevant in terms of population, geographic context, or topic, and non-primary publication types. During the full-text evaluation phase, studies were excluded if they did not directly involve service users—a central criterion of our review—as well as those with inappropriate study designs, non-pertinent outcomes, or samples that could not be disaggregated by ethnic group. The final reduction concerned the assessment of methodological quality, conducted using the Joanna Briggs Institute systematic review appraisal tool, with the exclusion of studies that did not meet a minimum threshold of methodological rigor. We employed a descriptive analysis approach to examine the selected documents [36]. This process involved systematically examining the data and extracting relevant information, in line with the principles outlined by Rapley [37] and the PRISMA-ScR guideline by Tricco et al. [38].

A standardized data extraction form was developed to capture key information from each study, including study characteristics (author, year, country, study design), participant characteristics (cultural groups, age, gender), mental health service context, key findings related to perceived discrimination and barriers to access, and recommendations for practice or policy. While formal quality assessment is not always conducted in scoping reviews, we chose to assess the methodological quality of included studies to provide context for the interpretation of findings.

The articles were expanded and categorised using an Excel spreadsheet. Several columns were created to divide the key data according to the country of study, the prevalent population, the ethnic minority population, the age of the people involved, the type of clinical service considered, the type of article considered, the methodology used by the authors, the results, the clinical implications, the discussion and the limitations of these studies (See Table 1).

Given the heterogeneity of the included studies, we adopted a descriptive synthesis approach. Descriptive analysis was used to identify and categorize key concepts and themes across the included studies, focusing on the user perspective of ethnic minorities in accessing mental health services and based on the aims of this research.

Throughout the data collection and analysis process, we maintained detailed records of our decisions and any deviations from the protocol to ensure transparency and reproducibility of our scoping review.

Data extraction was independently conducted by two authors following a predefined protocol. An Excel spreadsheet was used to record the essential elements of each study, including study characteristics, population details, methodology, and main findings. The extracted data were subsequently processed, and discrepancies were resolved through discussion and consensus. In cases where disagreements persisted, a third reviewer was consulted for the final decision. The extracted elements include authors, country, data collection period, sample size, research design, sampling method, and response type. The risk of bias was assessed using the Joanna Briggs Institute tools(version 2020) for cross-sectional studies, each comprising eight to ten response criteria. Overall, a “low” risk of bias was obtained.

The findings are organised around two interconnected macro-categories: linguistic and communicative barriers and organisational and economic barriers to mental health service access among ethnic minorities in Europe. Across both domains, a consistent pattern emerges: barriers rarely operate in isolation but interact and reinforce one another, producing cumulative disadvantage that systematically excludes minority populations from timely and appropriate care. These barriers do not, however, affect all minority individuals equally: their combined effects are systematically shaped by the intersection of ethnicity with gender, legal status, socioeconomic position, and generational status. In the sections that follow, we highlight specific instances in which an intersectional reading meaningfully shifts the interpretation of the findings (See Table 2).

### 3.1. Linguistic and Communicative Barriers

The reviewed literature consistently identifies language as one of the most pervasive obstacles to mental health service access, though its effects extend well beyond simple lexical misunderstanding. Four interconnected mechanisms characterise this barrier: linguistic distance as a marker of social exclusion, practical difficulties in everyday healthcare navigation, qualitative inadequacy of interpreting services, and the gap between Western medical language and cultural models of distress.

Linguistic distance has been operationalised and quantitatively measured in a multicentre European case–control study, which found that individuals whose mother tongue is distant from the host country language face an almost twofold probability of developing a first episode of psychosis (OR 1.94). Crucially, adjusting for linguistic distance reduced ethnic disparities in psychosis risk to non-significance, suggesting that language operates not merely as a practical barrier but as a genuine marker of sociocultural exclusion and psychosocial disempowerment [44]. This effect was particularly pronounced among first-generation migrants, while socioeconomic disadvantage became more prominent in subsequent generations. Complementary evidence highlights the complexity of linguistic acculturation: Pakistani and Indian migrants fluent in English showed depression rates comparable to British natives, yet paradoxically higher rates of anxiety, indicating that language acquisition does not uniformly confer psychological protection [40].

An intersectional reading of these findings is revealing: the protective effect of language acquisition appears to function differently depending on gender and socioeconomic position. Women from South Asian communities with greater English proficiency may still face culturally specific stressors—including family expectations, restricted mobility, and domestic roles—that sustain anxiety independently of linguistic integration. Interpreting language acquisition as straightforwardly protective risks obscuring how ethnicity and gender jointly shape mental health trajectories in ways that aggregate data conceal.

At the level of everyday healthcare navigation, qualitative findings from Polish migrants in Norway illustrate how linguistic barriers translate into concrete consequences—inability to understand written communications, avoidance of healthcare interactions, and reliance on informal strategies such as fellow nationals as translators or automatic translation applications [43]. In the context of British psychological services, the language barrier proved particularly acute in psychotherapy, where extended and nuanced conversation is required; nonetheless, English fluency alone did not fully account for disparities in service use between migrants and natives, pointing to the operation of additional factors beyond language per se [39].

The quality of interpreting services emerged as a critical and frequently neglected dimension. Research across intellectual disability and perinatal mental health contexts documented consistent qualitative failures: inability to distinguish between South Asian languages, interpreters claiming competence across multiple languages without possessing it, poor familiarity with mental health terminology, and the consequent disruption of the therapeutic relationship [51]. Rather than functioning as a tool for inclusion, inadequate interpreting services risk becoming a source of further clinical harm. Availability itself was found to be inconsistent, with marked variation in whether interpreter access was offered at all [51]. The communicative gap between Western medical frameworks and cultural models of distress represents a structurally distinct but equally significant barrier. Among Black populations in the United Kingdom, conceptualisations of mental illness linked to spiritual beliefs—including obeah, juju, and possession—rendered communication with clinicians trained exclusively in biomedical frameworks particularly difficult [42]. Low mental health literacy further compounded this gap, with patients struggling to articulate their experiences in medical language while professionals lacked the cultural competence to engage with alternative explanatory models [41,51]. Across contributions, what emerges as decisive is not practitioner ethnicity per se, but the capacity to demonstrate genuine curiosity, adapt communicative approaches, and avoid assumptions—qualities identified as central to culturally responsive care regardless of background [42,51].

### 3.2. Organisational and Economic Barriers

Structural, institutional, and socioeconomic factors interact to produce systematic inequalities in access, forming what the literature describes as a cycle of structural exclusion particularly well-documented among Black African and Black Caribbean populations in the United Kingdom. In this cycle, stigma generates distrust of services, leading to under utilisation and delayed help-seeking, which produces access only in states of acute crisis, resulting in coercive interventions that in turn reinforce stigma and distrust. The quantitative magnitude of this dynamic is striking: Black African populations face a twofold excess risk of short-term detention and a fourfold excess risk of compulsory treatment compared to white patients, with service access occurring disproportionately through police and criminal justice pathways rather than primary care [42].

An intersectional analysis substantially deepens this interpretation: the overrepresentation of Black African and Black Caribbean individuals in coercive pathways cannot be attributed solely to ethnicity. It reflects the accumulation of racialised institutional mistrust with socioeconomic marginalisation, low GP registration rates, and—for undocumented or recently arrived migrants—precarious legal status that actively discourages contact with statutory services. For Black women specifically, the intersection of race and gender may generate additional barriers, including culturally specific stigma around maternal mental health and reluctance to engage with services perceived as carrying child protection risks. Viewing these findings through an intersectional lens shifts the interpretive emphasis: coercive access is not a failure of individual help-seeking behaviour but a predictable outcome of overlapping structural disadvantages that converge differently depending on the specific combination of social positions involved.

Structural barriers to primary care access operate as a critical gatekeeper. Low GP registration—itself a product of administrative complexity, unfamiliarity with health systems, and temporary residency status—was identified as a primary determinant of reduced engagement with psychological services, since GP referral constitutes the main access channel to IAPT in the United Kingdom [39]. Among Polish migrants in Norway, the temporary identification number assigned to short-term residents created ambiguity about entitlement to GP registration, while unexpected healthcare costs and unfamiliarity with the gatekeeper model of specialist referral further discouraged engagement and promoted transnational service use in countries of origin [43]. A cohort study confirmed that migrants residing in the United Kingdom for fewer than ten years use IAPT services at a rate 0.4 times lower than UK-born individuals, a disparity that remained stable after adjustment for clinical severity, socioeconomic status, and adverse life events—excluding the possibility that lower need explains the gap [39].

Socioeconomic disadvantage functions both as a direct barrier to service access and as an independent risk factor for mental disorder, creating a self-reinforcing cycle of cumulative disadvantage. Among African-Caribbean migrants with schizophrenia in the United Kingdom, 80% were unemployed—twice the rate of Asian or white cases [40]. Indicators of socioeconomic disadvantage, including low parental socioeconomic status, educational attainment, relational isolation, and precarious living conditions, were associated with a significantly elevated psychosis risk (OR 1.54) [44]. Among South Asian caregivers of individuals with intellectual disabilities, unmet needs were markedly higher than among white caregivers (76% vs. 59%), with service use positively associated with higher family income and longer residence in the host country [51]. The differential access to talking therapies—frequently inaccessible without adequate financial resources—was explicitly articulated by participants: *“the knowledge of the alternatives might be there, but the access to them is not”* [59]. Poor knowledge of available services and cultural inadequacy constitute further and pervasive organisational barriers. Across perinatal mental health, intellectual disability, and community mental health contexts, low service awareness was identified as a primary obstacle, compounded by the absence of active outreach to minority communities [41,51]. Among South Asian caregivers, 94% identified lack of knowledge as the main barrier, with awareness of specialist services substantially lower than that of generic services. The cultural inadequacy of services—manifested in poor practitioner competence, non-representative staff composition, and the perception of services as designed for and by the white majority—was found to discourage engagement independently of objective service quality: *“if the community perceives that the facility is not really sensitive to my needs, no matter how good it is, I will not attend it”* [59]. This finding underscores that cultural responsiveness is not an optional enhancement but a structural precondition for equitable access.

## 4. Discussion

### 4.1. Summary of Principal Findings

This review identifies two interconnected domains of barriers systematically limiting mental health service access among ethnic minorities in Europe: linguistic and communicative barriers, and organisational and economic barriers. Across both domains, a consistent pattern emerges in which structural disadvantage, cultural incongruence, and institutional mistrust interact to produce cumulative inequalities in access and quality of care. From an interactionist perspective, these barriers are not fixed properties of individuals or systems but are actively constructed and reproduced through repeated encounters between minority service users and healthcare institutions—encounters in which asymmetries of symbolic power shape whose meanings and interpretations are recognised as valid [22,23]. Critically, the evidence suggests that under-utilisation of services cannot be attributed to lower need or individual reluctance alone—it reflects the operation of structural forces that precede and shape individual help-seeking behaviour [60,61]. A further cross-cutting finding concerns the ambivalence of access itself: increased contact with mental health services has not always been associated with benefit for minority populations, particularly where coercive practices predominate, raising important questions about the quality and cultural legitimacy of care, not only its availability [62].

### 4.2. Linguistic and Communicative Barriers—Comparison with Existing Literature

Language proficiency emerges across the reviewed evidence as a critical and multidimensional barrier, consistent with a broad body of prior research. Interpreters and translators are frequently absent from services, minorities may lack the communication skills required to navigate clinical encounters, and health and psychological terminology is often unfamiliar, making it difficult for individuals to articulate their own experiences [63,64]. Poor access to linguistically appropriate services has been documented across multiple contexts, including among children and young people from ethnic minorities, where lack of understanding of mental health problems, absence of information about services, and distrust of healthcare providers emerged as primary obstacles to help-seeking [65]. Among immigrants and refugees, language barriers intersect with stigma and unfamiliarity with mainstream mental health services, compounding difficulties in access and utilisation [66]. These findings are consistent with the existing literature, which demonstrates that linguistic barriers do not only affect communication but also the construction of meaning within the therapeutic relationship itself.

This communicative asymmetry has deep theoretical roots. Drawing on Salvini’s symbolic interactionism [24], meanings are continuously revised through social interaction rather than being static or predetermined. When renegotiation of meaning takes place in a language other than one’s own, this process may be fundamentally compromised. Critically, the interactionist framework illuminates how clinical encounters themselves become sites of symbolic negotiation: when the symbols and categories used by healthcare providers do not align with those of minority service users, the interaction risks producing not mutual understanding but mutual misrecognition—with significant consequences for diagnosis, treatment engagement, and therapeutic alliance [25]. As Berger and Luckmann [67] argued, reality is socially constructed through human interaction, with language as the primary medium of co-construction: the absence of a shared language between service users and providers therefore does not merely create a practical obstacle but undermines the very process through which trust and therapeutic alliance are built. If certain emotions are accessible primarily through one’s mother tongue [8], the assumption of intercultural equivalence in assessment and treatment requires critical scrutiny.

Healthcare workers who collect demographic data and recount symptoms often do so in a superficial manner, with errors in data collection and a tendency to record difficulties using terminology peculiar to the majority population rather than reflecting the person’s own narrative [68]. As Goffman [69] noted, even professionals who maintain a neutral predisposition inevitably apply their own interpretative systems and linguistic frameworks, which may not align with those of minority service users. Furthermore, as Kirmayer and Ryder [6] highlight, the process of renegotiation of meaning often does not take place, generating attributions of significance to personal experiences that ethnic minorities already struggle to articulate. Improving this dimension of care requires not only the provision of interpreters but investment in culturally and linguistically appropriate service delivery models built on collaboration between mainstream mental health services and community organisations [61,66].

### 4.3. Organisational and Economic Barriers—Comparison with Existing Literature

Socioeconomic disadvantage and organisational complexity constitute a second and equally pervasive domain of barriers, consistent with prior European evidence. Social and economic disadvantage tends to persist over time, with differences between ethno-cultural minority groups becoming increasingly entrenched, producing heightened risk for poor mental health among individuals with minority heritage [70]. Chronic exposure to discrimination has an incremental negative long-term effect upon mental health and the probability of seeking help [15]. Levecque and Van Rossem [71] observed that in most European countries, immigrants experience more depressive symptoms than the native-born population, primarily reflecting their poorer socioeconomic position, while Malmusi [72] further demonstrated that health inequalities are greater in countries with more restrictive integration policies. In the United States context, affordability and lack of insurance coverage have been identified as the most prevalent barriers to healthcare access among individuals with mental health challenges, with all five dimensions of healthcare access—availability, accessibility, accommodation, affordability, and acceptability—found to interact in producing unmet need [73]. Unawareness of available services was described across studies as the greatest single barrier to accessing help [30,41,51], a finding replicated among children and young people from ethnic minorities, where lack of information about services and cultural expectations around mental resilience were identified as critical obstacles [65]. However, a lack of awareness does not fully explain service under utilisation: for some communities, reduced use of mental health services may reflect a deliberate and historically grounded choice, shaped by experiences of discrimination or cultural devaluation within those very services [74,75,76,77]. From an interactionist standpoint, this pattern of avoidance can be understood as a rational adaptive response to repeated negative interactions with institutional services—interactions in which minority service users have learned that their symbolic frameworks, explanatory models, and cultural meanings are systematically devalued or ignored [26,27]. Among Black Americans, racism has been identified as a direct cause of mistrust in mental health service systems, with fear of discrimination—based on both race and mental illness—found to be particularly pronounced among more highly educated individuals [78]. The disproportionate rates of involuntary admissions and coercive treatments documented among people of African and Caribbean descent suggest that, for some groups, increased contact with mental health services has been associated with harm rather than benefit [62,74,79,80]. Mistrust of institutional services should therefore not be interpreted solely as an irrational barrier to be overcome, but as a historically rooted and legitimate response to institutions whose practices have not always respected the interests of minority communities [5,81]. Addressing under utilisation thus requires ensuring that services are themselves worthy of the trust they seek to earn [82,83,84]. Minorities tend to turn first to individuals belonging to their own culture, traditions, and family or peer networks. Family members frequently report negative experiences with healthcare facilities, generating a mechanism of distrust and powerlessness transmitted across generations [85]. Even when members of ethnic minority groups believe the system may help them, shame and fear of ostracism from family or community members may prevent them from seeking help [13]. Cultural stigma operates across multiple dimensions—structural, affiliative, public, and self-stigma—with minority groups consistently reporting higher levels of public and self-stigma than majority populations, and with specific cultural beliefs, family dynamics, and fears of reinforcing stereotypes shaping help-seeking behaviour in distinct ways across groups [86]. Religious or traditional values such as collectivism and conformity may further influence the way minorities perceive symptomatic behaviour and the extent to which they feel personally affected by it [6].

### 4.4. Cross-Cutting Interactions and Theoretical Implications

Across both domains, barriers rarely operate independently: linguistic distance amplifies the effects of organisational complexity; socioeconomic disadvantage reduces access to interpreting services and talking therapies alike; institutional mistrust is reinforced by culturally inadequate encounters, which are themselves partly a product of the communicative asymmetry described above.

These interactions produce a cycle of cumulative disadvantage that cannot be addressed through single-domain interventions. Among adolescents from ethnic minority backgrounds, this multi-level interaction is particularly evident: individual-level factors such as symptom severity and cultural stigma interact with family-level dynamics—including parental stress, migration-related stressors, and low mental health literacy—and with structural factors such as low household income and limited school-based support, collectively shaping pathways into care [60]. The interactionist framework proves particularly generative here: it directs analytical attention not only to individual characteristics or structural conditions in isolation, but to the interaction itself as the unit of analysis—the specific moments in which meanings are negotiated, trust is built or eroded, and pathways into or away from care are constructed [28,29]. Barriers and facilitators, from this perspective, are not static features of individuals or systems but emergent properties of repeated interactions across time and context. This has direct implications for intervention design: changes in the symbolic quality of clinical encounters—in the degree to which minority service users feel genuinely heard, understood, and respected—may be as consequential as structural reforms in determining whether care is accessed and sustained.

A further cross-cutting issue concerns the intercultural equivalence of psychiatric care itself. The literature reviewed risks treating language barriers as technical problems amenable to practical solutions—the provision of interpreters, translation of materials, training of culturally competent professionals. Whilst these measures are undoubtedly valuable, they do not address a more fundamental epistemological challenge: the evidence that emotional experience is not culturally or linguistically neutral, and that access to one’s emotional life is often deeply tied to one’s mother tongue [8,87].

We advance here an interpretive proposition that goes beyond what the included studies directly demonstrate, but which we consider analytically warranted: therapeutic conversations conducted in a second language, or mediated by an interpreter, may therefore not only be less effective, but may also actively hinder the emotional processing central to treatment. Similarly, as an analytical inference rather than an empirical conclusion drawn from the reviewed literature, it cannot be taken for granted that diagnostic categories and therapeutic models developed in Western, English-speaking contexts retain equivalence when applied across linguistic and cultural boundaries: the concept of a symptom, the expression of distress, and the therapeutic relationship are all shaped by the cultural and linguistic systems in which they are embedded [6]. These propositions are offered as conceptual contributions intended to orient future empirical inquiry, rather than as findings directly supported by the scoping review. A genuinely inclusive approach requires services to be provided in multiple languages, to ensure that therapies are culturally appropriate—including non-Western approaches such as yoga, meditation, and complementary therapies—and to pursue more robust data collection across ethnic groups to target research and service improvement effectively [61].

### 4.5. Limitations

Several limitations of this review warrant acknowledgement. The heterogeneity of study designs, populations, and outcome measures across the included literature limits the comparability of findings and the extent to which conclusions can be generalised. The majority of evidence derives from the United Kingdom and Northern Europe, which may not fully reflect the experiences of minorities in other European contexts, particularly Southern and Eastern Europe where migration patterns and health system structures differ substantially. Furthermore, the category of ethnic minority encompasses considerable internal diversity—differences by minority group, gender, education level, generation, and cultural orientation are documented in the literature [12,60]—and aggregate findings may obscure important variation within and across groups. The relative scarcity of studies focused on children, young people, and adolescents from ethnic minorities in European contexts represents a further gap, given emerging evidence that barriers to access manifest in developmentally specific ways in younger populations [65]. Publication bias towards studies documenting barriers, rather than facilitators, of access may further limit the representativeness of the evidence base.

### 4.6. Practical, Theoretical and Policy Implications

The findings carry significant implications across three levels.

At the practice level, services must move beyond the provision of interpreters as a primary response to linguistic diversity, towards genuinely culturally responsive care that recognises the role of language in shaping emotional experience, meaning-making, and therapeutic alliance. Communication styles and attitudes among healthcare personnel have been identified as primary obstacles to quality service access [88], and healthcare workers must be supported to engage with minority patients’ own narratives rather than imposing majority-population frameworks [68]. An interactionist approach to clinical training would direct practitioners’ attention to the quality of symbolic exchange within each encounter—cultivating the capacity to recognise and suspend their own interpretative frameworks, and to create the conditions under which minority service users can articulate their experiences in their own terms [22,23]. Mental health practitioners should review current approaches to assessment and treatment through a destigmatising and culturally sensitive lens, actively promoting open and inclusive practices [61]. The involvement of ethnic community members, religious leaders, and family networks as active partners in service delivery represents a further evidence-informed strategy for improving engagement [82], as does investment in cultural brokers and interpreters equipped with mental health training [66]. Culturally tailored stigma reduction interventions—incorporating indigenous labels, explanatory beliefs, family involvement, and alternative treatments—represent a promising but underdeveloped area requiring further investment [86].

At the theoretical level, the findings call for a more radical rethinking of psychiatric care premises than the cultural adaptation of existing models alone can provide. A genuine paradigm shift is required—one that questions not only the ways in which services are delivered, but also the universality of the conceptual frameworks on which they are based, and that takes seriously the evidence that emotional experience and its therapeutic processing are deeply embedded in the linguistic and cultural systems within which they occur. For Black communities in particular, this requires mental health systems to explicitly confront racism—both in its historical dimensions and in its contemporary manifestations within services—and to engage in critical self-reflection at the individual level and racial equity analysis at the organisational level [62,78].

At the policy level, the evidence that health inequalities are greater in countries with more restrictive integration policies [72] underscores that mental health equity cannot be achieved through health sector interventions alone. Public mental health services should adopt both formal and informal integration strategies, and policy frameworks must address the structural determinants—unemployment, precarious housing, restricted legal status—that generate and sustain mental health disparities. Reducing disparities in access to mental health care is a necessary but insufficient goal: a parallel commitment to transforming the quality, safety, and cultural legitimacy of the care provided is equally required, so that greater access translates into genuinely beneficial and equitable outcomes rather than greater exposure to coercive or culturally inappropriate interventions [83,89]. Policymakers and service commissioners must acknowledge the need for more inclusive mental health practices and ensure that data collection across ethnic groups is sufficiently robust to support targeted research and service improvement [61].

### 4.7. Future Research Directions

Future research should prioritise several underexplored areas. First, the mechanisms underlying ethnic differences in help-seeking require more systematic investigation, moving beyond descriptive accounts of barriers towards explanatory models that account for the interaction of structural, cultural, and individual factors [12]. An interactionist approach can offer valuable tools for understanding how health services are able to work on everyday interactions to reduce barriers and promote listening and sharing. Longitudinal and ethnographic studies on real clinical encounters would allow real-time observation of how trust is built or broken, and how service responses shape subsequent help-seeking pathways—providing concrete insights into how to intervene effectively at the level of micro-interaction [30,90].

Second, the intercultural equivalence of diagnostic categories and therapeutic models demands empirical scrutiny—research examining how distress is experienced, expressed, and processed across linguistic and cultural contexts would provide an important foundation for more epistemologically robust approaches to intercultural mental health care.

Third, longitudinal designs are needed to examine how barriers evolve across generations and in relation to changing integration contexts, given evidence that the relative weight of linguistic and socioeconomic factors shifts between first and subsequent generations [44].

Fourth, research should attend more carefully to within-group heterogeneity, examining how factors such as gender, education, legal status, and cultural orientation interact to shape access and outcomes across different minority communities [86].

Fifth, dedicated research on children, young people, and adolescents from ethnic minorities is needed, given the limited evidence base in this area and the specific developmental and systemic factors—including school-based pathways, parental gatekeeping, and culturally shaped symptom recognition—that shape their access to care [60,65].

Finally, intervention research evaluating the effectiveness of culturally adapted and community-based approaches—including the involvement of community members, peer support, informal networks, and anti-racist service reform—would substantially strengthen the evidence base for policy and practice [61,66,78].

## 5. Conclusions

This review identified two principal categories of barriers—linguistic/communicative and organisational/socioeconomic—that interact to systematically limit mental health service access among ethnic minorities in Europe. These findings underscore the need for systemic and culturally sensitive interventions that go beyond simply increasing service availability, addressing instead the structural and epistemological conditions that shape whether care is accessible, trustworthy, and genuinely beneficial.

At the practice level, services can be optimised by attending closely to personalisation, respect for individual needs, and cultural responsiveness in each service encounter. At the policy level, interventions should be informed both by their impacts on individuals and by a broader understanding of local communities, maximising benefits and minimising harms for all those affected. Critically, greater access must not be regarded as an end in itself: what matters equally is the quality, cultural appropriateness, and safety of the care provided. Monitoring of service use among migrant and ethnic minority populations must therefore be accompanied by ongoing assessment of whether care is genuinely perceived as beneficial and respectful, rather than coercive or culturally alienating [85]. Future research should prioritise the systematic evaluation of cultural adaptations—both in isolation and in combination—to identify which approaches increase effectiveness and for whom, with attention to cost analyses to inform future decision-making. The moderating role of acculturation warrants further investigation, as does the applicability of findings beyond the Western European contexts that dominate the current evidence base. Studies conducted in underrepresented settings—including the Italian context—would substantially strengthen the generalisability of conclusions in this field.

## Figures and Tables

**Table 1:** Study Selection and Characteristics.

	Author(s)	Year	Title	Country	Objective(s)
1	Bhavsar, V. et al. [39]	2021	The association of migration and ethnicity with use of the Improving Access to Psychological Treatment (IAPT) programme: a general population cohort study.	United Kingdom	To examine ethnic and migration-related differences in use of IAPT-based psychological treatment using a novel epidemiological dataset with linkage to de-identified IAPT records.
2	Bhugra D. [40]	2004	Migration and mental health.	United Kingdom	To distil existing information on how migration influences individuals mental state and how it determines help seeking as well as pathways to care.
3	Causier C. et al. [41]	2024	Experiences of help-seeking for severe mental health problems in young Pakistani women: A preliminary qualitative study.	United Kingdom	To understand how young Pakistani women and their parents make decisions to seek help for severe mental health problems, and the barriers and facilitators to accessing professional help.
4	Devonport, T.J. et al. [42]	2023	Systematic Review of Inequalities in the Mental Health Experiences of Black African, Black Caribbean and Black-mixed UK Populations: Implications for Action.	United Kingdom	To review the reported mental health of Black African Caribbean communities in the UK, determinants of mental health, and interventions to enhance their experiences of mental health services.
5	Czapka, E.A., Sagbakken, M. [43]	2016	Where to find those doctors?” A qualitative study on barriers and facilitators in access to and utilization of health care services by Polish migrants in Norway.	Norway	To identify the main barriers and facilitators experienced by post-accession Polish migrants in accessing and utilizing health care services in Norway.
6	Jongsma, H. et al. [44]	2021	Social disadvantage, linguistic distance, ethnic minority status and first-episode psychosis: results from the EU-GEI case–control study.	United Kingdom	Ethnic minority groups in Western countries face an increased risk of psychotic disorders. Causes of this long-standing public health inequality remain poorly understood. We investigated whether social disadvantage, linguistic distance and discrimination contributed to these patterns.
7	Healey, P. et al. [45]	2017	Cultural adaptations to augment health and mental health services: a systematic review.	Europe	To identify extant themes in the research regarding cultural adaptations across a broad range of health and mental health services and synthesized the most rigorous experimental research available to isolate and evaluate potential efficacy gains of cultural adaptations to service delivery.
8	Robertson J. et al. [46]	2019	What do we know about the health and health care of people with intellectual disabilities from minority ethnic groups in the United Kingdom? A systematic review.	United Kingdom	(1) To summarize what is known about the health status of those with intellectual disabilities from minority ethnic communities in order to document potential health inequalities and identify gaps in knowledge. (2) To provide a narrative synthesis of research relating to the physical or mental health care of people with intellectual disability from minority ethnic communities in order to provide potential directions for future research, policy and practice.
9	Malmusi D. et al. [47]	2014	Health inequalities in immigrant populations in Spain: a scoping review.	Spain	To know the influence of social determinants of health in the immigrant population in Spain and/or inequalities compared to the Spanish population.
10	Kieseppä, V. et al. [48]	2020	Immigrants’ mental health service use compared to that of native Finns: a register study.	Finland	To compare the intensity of psychiatric care, as an indicator of treatment adequacy, between natives and immigrants living in Finland.
11	Kolvenbach, S. et al. [49]	2018	Perceived treatment barriers and experiences in the use of services for obsessive–compulsive disorder across different ethnic groups: a thematic analysis.	United Kingdom	To identify and compare barriers that parents from different ethnic groups face when accessing specialist services for obsessive–compulsive disorder (OCD) for their children.
12	Lindert J. et al. [50]	2008	Mental health, health care utilisation of migrants in Europe.	Europe	To give an overview on (i) prevalence of mental disorders; suicide; alcohol and drug abuse; (ii) access to mental health psychosocial care facilities of migrant in the European region, and (iii) utilisation of health and psychosocial institution of these migrants.
13	Conneely M. et al. [51]	2023	Exploring Black and South Asian women’s experiences of help-seeking and engagement in perinatal mental health services in the UK.	United Kingdom	To answer two questions: how do Black and South Asian women experience (1) access to perinatal mental health services and (2) care received from perinatal mental health services.
14	Malmusi D. et al. [52]	2017	Inequalities by immigrant status in depressive symptoms in Europe: the role of integration policy regimes.	Europe	To study whether country integration policy models were related to inequalities by immigrant status in depressive symptoms in Europe.
15	Markova V. et al. [53]	2020	Immigration, acculturation, and preferred help-seeking sources for depression: Comparison of five ethnic groups.	Norway	To understand potential ethnics differences in preferred help-seeking sources for depression in Norway, and how such preferences relate to acculturation orientation.
16	Leijten, P. et al. [54]	2016	Ethnic differences in problem perception: Immigrant mothers in a parenting intervention to reduce disruptive child behavior.	Netherlands	To examine ethnic differences in problem perception by mothers engaged in a parenting intervention.
17	Kieseppä et al. [55]	2021	Depression and anxiety disorders among immigrants living in Finland: Comorbidity and mental health service use.	Finland	The aim of this study is to compare the differences in the background characteristics and comorbidity between immigrants and native Finns diagnosed with depression and/or anxiety disorders in specialized health care, and to compare the intensity of mental health service use.
18	Henderson, R.C. et al. [56]	2015	Mistrust of mental health services: ethnicity, hospital admission and unfair treatment.	United Kingdom	To explore the role of psychiatric admission, diagnosis and reported unfair treatment in the relationship between ethnicity and mistrust of mental health services.
19	Ayub, R., Macaulay, P.J.R. [57]	2023	Perceptions from the British Pakistani Muslim community towards mental health.	United Kingdom	To explore the perceptions of the British Pakistani Muslim community towards mental health and barriers towards seeking treatment.
20	Alam S., O’Halloran S., Fowke A. [58]	2024	What are the barriers to mental health support for racially minoritised people within the UK?	United Kingdom	(1) To better understand the needs of the population and factors which impact wellbeing. (2) To consider the muti-faceted barriers to access mental health support, such as CBT, and how to address these. (3) To unpack what stigma means (internally and externally) for men in the population. (4) How to support low- and high-intensity CBT practitioners to better work therapeutically to support Bangladeshi men.

**Table 2:** Summary Table—Key Barriers and Evidence.

Barries	Population	Key Finding	Reference
Linguistic distance	Multi-ethnic, Europe	OR 1.94 for first-episode psychosis	[44]
Language & psychotherapy access	Migrants, UK	Reduced IAPT use (rate 0.4×); fluency insufficient	[39]
Everyday language barriers	Polish migrants, Norway	Informal coping strategies; inconsistent interpreter access	[43]
Interpreting service quality	South Asian, perinatal	Misattribution, poor competence, therapeutic disruption	[51]
Cultural models of distress	Black populations, UK	Spiritual conceptualisations incompatible with biomedical framework	[42]
Structural exclusion cycle	Black African/Caribbean, UK	2× detention risk; 4× compulsory treatment risk	[42]
GP registration & system navigation	Migrants, UK/Norway	Low registration as gateway barrier	[39,43]
Socioeconomic disadvantage	African-Caribbean, UK	80% unemployment; OR 1.54 psychosis risk	[40,44]
Unmet needs & income	South Asian caregivers	76% unmet needs vs. 59% white caregivers	[51]
Service awareness & cultural inadequacy	Multiple minorities, UK	94% unaware of specialist services; perception of inadequacy deters access	[41,51,59]

## Data Availability

No new data were created or analysed in this study. Data sharing is not applicable to this article.
